# Studying information recurrence, gatekeeping, and the role of communities during internet outages in Venezuela

**DOI:** 10.1038/s41598-021-87473-8

**Published:** 2021-04-14

**Authors:** Pamela Bilo Thomas, Emily Saldanha, Svitlana Volkova

**Affiliations:** 1 University of Notre Dame, Notre Dame, USA; 2 Pacific Northwest National Laboratory, Richland, USA

**Keywords:** Information theory and computation, Mathematics and computing, Computational science, Computer science

## Abstract

Many authoritarian regimes have taken to censoring internet access in order to stop the spread of misinformation, restrict citizens from discussing certain topics, and prevent mobilization, among other reasons. There are several theories about the effectiveness of censorship. Some suggest that censorship will effectively limit the flow of information, whereas others predict that a backlash will form, resulting in ultimately more discussion about the topic. In this work, we analyze the role of communities and gatekeepers during multiple internet outages in Venezuela in January 2019. First, we measure how critical information (e.g., entities and hashtags) spreads during outages focusing on information recurrence and burstiness within and across language and location communities. We discover that information bursts tend to cross both language and location community boundaries rather than being limited to a single community during several outages. Then we identify users who play central roles and propose a novel method to detect gatekeepers—users who prevent critical information from spreading across communities during outages. We show that bilingual and English-speaking users play more central roles compared to Spanish-speaking users, but users inside and outside Venezuela have similar distribution of centrality. Finally, we measure the differences in social network structure before and after each outage event and discuss its effect on how information spreads. We find that with each outage event social connections tend to get less connected with higher mean shortest path, indicating that the effect of censorship makes it harder for information to spread.

## Introduction

Of internet users, 38% live in countries where social media or online messaging has been blocked in the previous year^1^. Countries block access to the internet for political, social, or national security reasons^2^. With many regimes around the world resorting to censorship^3^, including long-term continuous censorship in China^4,5^, and short-term censorship events in Iran in 2017, Pakistan, Thailand, and Venezuela^6–8^, it is important to understand and explicitly measure how human communications and social network structure change as a result of an external shock such as a censorship event, and the role of language and location communities, information recurrence patterns, and gatekeeping during such disruptive events intended to affect information diffusion.

In this work, we answer the above questions by analyzing Twitter activity related to the recent internet outage events occurring in Venezuela on January 21, 2019^7^, January 23, 2019^6^, and January 26–27, 2019^8^ affecting much of the southwest and east of the country during ongoing political protests. The first two outages primarily included disruptions to social media access—Twitter, Instagram, YouTube, while the third was a total internet outage. Most Venezuelans access the internet over one state-run internet provider CANTV^9^. Over 12 million Venezuelans use social media and 3 million of them are active Twitter users^10^. The cause of the outages remains unclear but are technically consistent with controls used to filter internet content and restrict access to the internet in the country. These outages happened during a period of political turmoil over the disputed presidency and coincided with the dissemination of anti-government videos online and speeches given by Juan Guaidó^11^. The disruptions in Periscope, YouTube, Facebook and Instagram on January 27 are likely to have impacted audience reach and viewership of live-streamed speech by Juan Guaidó on that day. During political protests in 2018, 52 websites were blocked by Venezuelan internet providers^9^.

Our science objectives aim to advance understanding of information diffusion during censorship events, including the evolution of social structure and the role of social groups and communities as well as individuals in facilitating or hindering the spread of information. In this work, we define a community as a group of Twitter users who belong to a language or location group. We focus on five separate communities: Spanish speakers, English speakers, Spanish and English (bilingual) speakers, Venezuelans, and Non-Venezuelans. An individual can belong to both a language community and a location community. To achieve these objectives, we focus on answering the following research questions:

- How do external outage events affect social network structure? What does the social structure look like before, during, and after an internet censorship event?
- How does discussion of specific entities, such as political figures, hashtags, and social media companies, recur and evolve over time during repeated outage events?
- Which users serve in central roles connecting different groups and communities (e.g., across languages and geolocations) during outages?
- Which users function as gatekeepers across communities, determining whether specific information will spread to users to that community during outages?

## Previous work

### Social network structure and information diffusion

Social networks play a significant role in how information spreads^12,13^ and how users adopt social behaviors^14^. As has been shown in the literature, community structure^15^ and network assortativity can effect spread patterns and be good predictors of the strength of diffusion of memes on Twitter^16^. Strong communities enhance social reinforcement^17^ and therefore enhance local information diffusion, while weak community structure enhances the global spread. Moreover, it has been demonstrated that information spreads further and faster across cluster-lattice networks^18^. Hubs and degree distribution have been studied extensively due to their role in epidemic spread^15^. Hubs, however, have been shown to have contrasting effects on information diffusion^19^. Finally, it has been shown that network density is positively related to information diffusion^20^.

To the best of our knowledge, this work is the first to study how external outage events affect social network structure. Our analysis reveals novel insights into properties of the social network structure before, during, and after multiple internet censorship events.

### Information recurrence

Information recurrence is a common pattern on social media platforms^21^. Information that is highly popular and spreads quickly is more likely to recur in the future^22^. Additionally, information that is moderately appealing can recur in the future, as information that has already spread widely in the population will be more likely to not be reshared by those individuals who have already seen it. In addition, other results show that false rumors are more likely to recur in the future in comparison to true rumors^23^.

Unlike earlier work that primarily focuses on recurring information cascades, we study how the spread of specific units of information recur in an environment with repeated and ongoing external shocks – specifically internet outages. We identify units of information (e.g., named entities and hashtags) and study the patterns of recurrence of these units of information to identify when and where bursts in the sharing of that information occur, with particular interest in comparing when bursts are isolated to individual communities and when they spread across the larger social network.

### Gatekeeping

In general, a gatekeeper is a person or entity who has the discretion to control the flow of information^24^. According to some definitions, a gatekeeper can be an individual or organization that consumes content which contains a variety of information but tends to produce content aligning with only one side^25^. Some studies have called social media managers gatekeepers, since they determine what information their followers will see^26^. A user’s capacity for functioning as a gatekeeper is partially determined by their level of influence^27–30^, with possible measures of influence including number of followers, page rank, number of retweets, or number of mentions. However, these measures do not always tell a consistent story, as users who rank highly in one measure of influence can do poorly in another^31,32^.

One study found that gatekeepers with higher levels of trust will be more successful in spreading their messages^33^. Another study concluded that if protests are taking place in developing countries, many use social media to spread information about what is happening to a Western audience with the purpose of causing change in their own countries^34,35^. Others found that the use of political hashtags, number of social connections of users, and positions of users within social networks are more important than network topology for viral spread^36^.

Another line of work focuses on identifying and quantitatively measuring language bridges^37–39^, including sociolinguistic studies that focus on understanding bridging topics^40^. They not only analyze connectivity of multilingual communities on Twitter^41^, but also investigate dynamics of language change online^39^.

We build on these earlier efforts by studying the role that users can play as gatekeepers in determining information flow between communities, specifically focusing on language- and location-based groups, in the context of repeated and ongoing outage events that have the potential to restrict the flow of information.

## Data

In order to study the information diffusion patterns related to the repeated internet outage events in Venezuela, we leverage Twitter data collected using the public Twitter API covering the date range from January 14 to February 9, 2019, spanning the outage events occurring on January 21, January 23, and January 26–27, 2019. The data were collected using a query that contains popular hashtags from the time of the protests and internet outages as shown below:

(Internet OR censorship OR censura) AND (Venezuela OR Maduro OR #LasCallesSonDelChavismo OR #LealesSiempreTraidoresNunca OR #23E OR #23ene OR #23Enero OR #VenezuelaGritaLibertad OR #VenezuelaLibre OR @jguaido OR Venezuela has:geo OR Caracas has:geo).

This query was designed to collect discussions of the Venezuelan censorship activities, and the resulting data collection contains a total of 276,787 tweets by 148,662 unique users, with 205,811 retweets, 64,198 quoted tweets, and 7,822 replies. The raw JSON data was processed and important information needed for the analysis was extracted from the metadata: tweet content, tweet languages, tweet time, user-specified locations, etc. Mixed-method, interpretative statistical analysis was used to analyze the extracted data—tweets, users, languages, and locations—and answer the above research questions.

**Figure 1:**
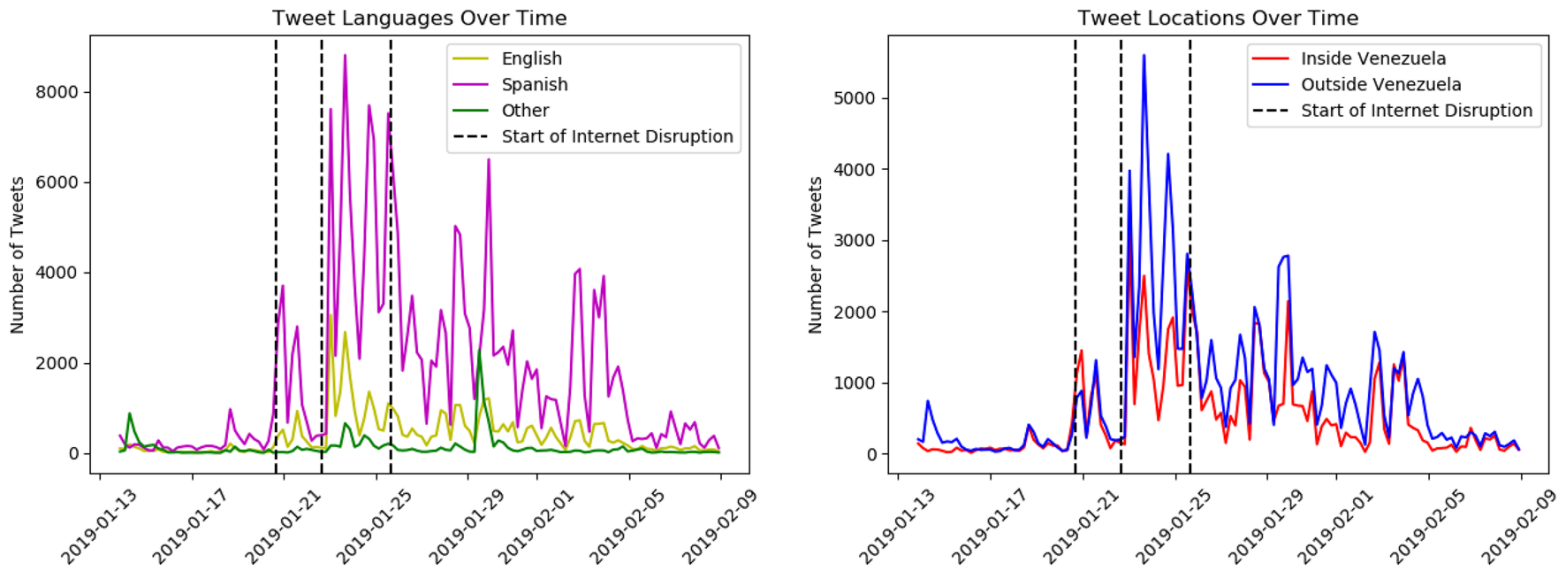
A breakdown of tweet counts by language (left) and user location (right) that we have collected using our Twitter query, with the three internet outage events marked by vertical dashed lines.

For our analysis we break down the user population into two location communities, inside and outside Venezuela, and two language communities, English and Spanish speakers. It is possible for users to belong to both the contrasted communities, although this is much more prevalent in the language-based communities. The distribution of tweets over time and across languages (English, Spanish, other) and locations (outside, inside Venezuela) is presented in Fig. 1. We observe that tweets continued to be observed from users within Venezuela, even during the internet outages. This could be because the outages may not have affected the entire country uniformly, continuously, or across all platforms. It is also possible that internet-savvy Venezuelans were able to get around the internet outages by using VPNs or other techniques..

## Methods

### Location assignment

Because a portion of our analysis focuses on location-based communities, we must determine which users are located inside and outside Venezuela. Twitter users have the option of setting their tweets to be location-enabled, in which case we would definitively know their location, but only a small portion of tweets in our sample have location enabled (1,121 out of 276,787 tweets). Due to this sparsity, we look at the listed locations of user Twitter profiles, which allows more complete coverage of the dataset. We define a tweet from inside Venezuela to be from someone who has “Venezuela,” “Caracas,” “Valencia,” “Maracaibo,” or “Barquisimeto” in their location profile. We define individuals who are tweeting from outside Venezuela as those who do not have any of the locations above listed in their profile and do not have ‘None’ as a location. We validate this approximation approach by comparing the countries assigned using the user profile to the geotagged tweet location for the 1,121 tweets which have a tagged location. We find that the country of the tweet agrees with the country in the profile 92% of the time and that the accuracy of the inside versus outside assignment is also 92%. Using the user profiles, approximately 23.5% of tweets from users were from individuals who could be determined to be inside Venezuela and 38.4% of the tweets were assigned to be from outside of Venezuela.

**Table 1:** Dataset statistics by language and location in terms of the number of tweets and users.

	Number tweets	Percentage tweets	Number users	Percentage users
Spanish	230,822	83.3%	99,881	67.1%
English	29,226	10.5%	26,518	17.8%
Venezuela	65,222	23.5%	27,004	18.1%
Not Venezuela	105,643	38.1%	59,794	40.2%

The dataset is skewed towards Spanish speakers and users that are outside of Venezuela.

### Language assignment

To assign the users to language communities, we observe the languages used in their individual tweets rather than using the Twitter-assigned default language. Twitter assigns default languages to users, but it is also possible for users to tweet in multiple languages. Twitter also assigns languages to tweets, which might be different than the default language of the user. We used this tweet-level language metadata to determine which language our users were tweeting in. Our data show 67.1% of users use Spanish as their primary Twitter language and 17.8% use English; however, 83.3% of the tweets are in Spanish and 10.5% are in English. For our analysis, we define a bilingual user as someone who tweets at least once in both Spanish and English, while English and Spanish speakers are those that tweet exclusively in those languages. We present detailed statistics for the number and percentage of tweets and users across locations and languages in Table 1.

### Measuring social interaction change during internet outages

In order to study the social connections between users during outage events, we constructed several social networks from our dataset based on the observed interactions between users by breaking down the data into different temporal subsets as well as analyzing the social network derived from the full set of data. Since it is unclear how long the internet was unavailable in Venezuela during each outage, we study the full day when the internet was disrupted for standardization purposes.

**Table 2:** Social network properties of the full Twitter data collection.

Network property	Value
Number of nodes	276,787
Density, $10^{-4}$	0.265
Number of connected components	3075
Maximum node degree	13,127
Average node degree	3.739
Mean shortest path length	4.384
Assortativity coefficient	0.0364
Average clustering coefficient	0.1458

### Social network construction details

We constructed networks of users where each edge corresponds to an interaction between users. Possible interactions include a user retweeting, replying to, and quoting another user. It is important to note that we do not have the follower information included in this dataset. Because the Twitter API does not distinguish the direct parent of a retweet (which may have been the original tweet or another retweet of that tweet), but only specifies the original root tweet, the social links that we infer due to the retweets will all point at the user of the original tweet. Therefore, this approximates the true social interaction network. The properties of the full social interaction network generated from this dataset can be seen in Table 2.

We see in Table 2 that our overall social network consists of many connected components; there are over 3,000 in our dataset. Meanwhile, the maximum node degree is 13,127, which is four times more than the average degree. Similar to many social graphs, a large number of nodes have a small degree, while a small number of nodes have a disproportionately high degree, signifying the scale-free, power-law nature of the degree distributions. On average, individuals in our network are separated by 4.3 hops. We observe an assortativity coefficient of 0.14, indicating that our network is disassortative. Further, we also see a low average clustering coefficient. These data indicate our network is fairly unconnected and many nodes exist in separate sub-networks from one another.

### How do external outage events affect social network structure?

To study changes in user connectivity during an internet outage, we created interaction networks for different subsets of the data. For each outage, we created two separate networks, one where each edge corresponds to connections that were observed during the outage but were not observed immediately before the outage occurred and one where each edge was a connection that was observed before the outage but not during it. We call these the “added connection” and “disappeared connection” networks, respectively. In this way, we observe which connections were newly formed during outages, possibly prompted by external events, and which connections were no longer observed during an outage event.

**Figure 2:**
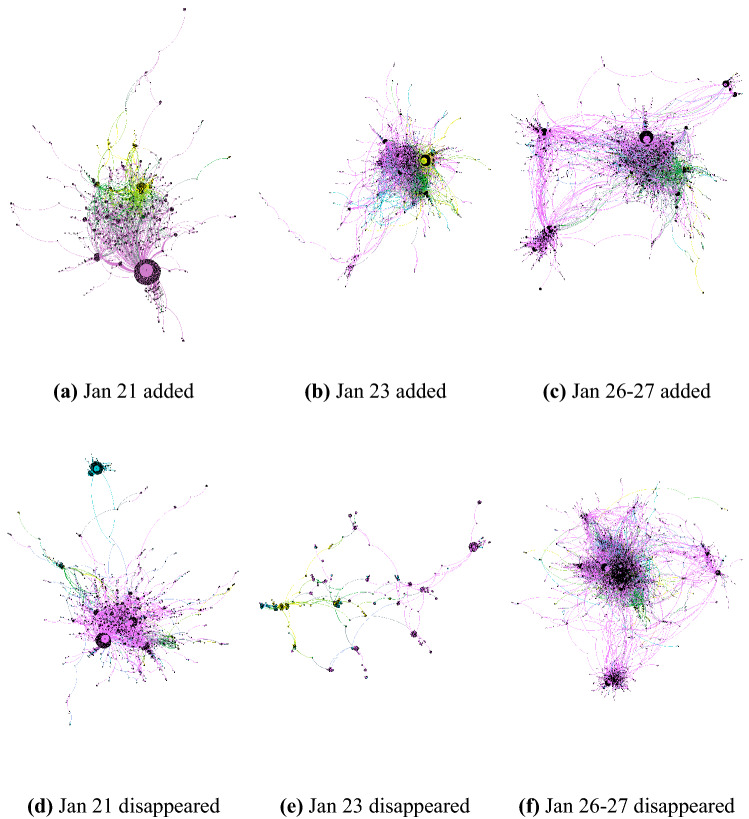
Network visualizations of the largest connected component of the added connections (top) and disappeared connections (bottom) in social interaction networks for each of the three internet outage events in Venezuela by language (Spanish—purple, English—yellow, bilingual—green, other—teal). We find that after every network outage, the added graph has longer mean shortest paths (3.342, 3.918, and 4.587), more connected components (158, 661, and 922), and a larger number of communities (205, 734, and 870), compared with the previous outage.

**Figure 3:**
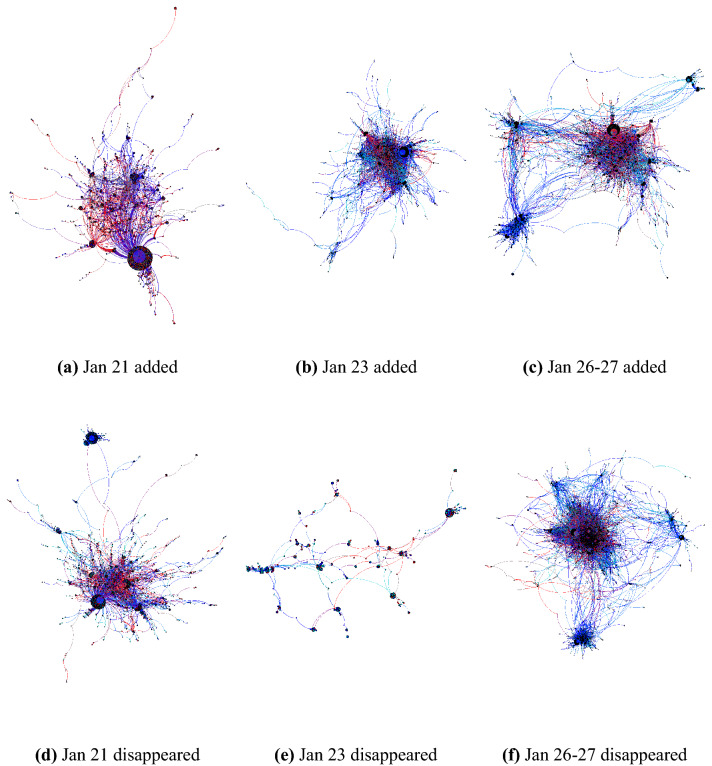
The same networks of added connections (top) and disappeared connections (bottom) as shown in Fig. 2 for each of the three internet outage events in Venezuela now colored by location (Venezuela—red, outside Venezuela—blue).

**Table 3:** Network properties of “added” and “disappeared” social connections before and during internet outages.

	Jan 21 Disappeared	Jan 23 Disappeared	Jan 26–27 Disappeared
Number of nodes	13,239	2550	41,660
Connected components	582	206	1365
Density, $10^{-4}$	1.69	8.67	0.652
Number of communities	626	225	1426
Maximum node degree	3456	399	5827
Average node degree	2.243	2.210	2.714
Mean shortest path length	4.313	6.123	4.928
Average clustering	0.060	0.075	0.079

	Jan 21 Added	Jan 23 Added	Jan 26–27 Added
Number of nodes	10,380	28,826	22,779
Connected components	158	661	922
Density, $10^{-4}$	2.31	0.90	1.11
Number of communities	205	734	870
Maximum node degree	6157	11,785	5450
Average node degree	2.399	2.607	2.544
Mean shortest path length	3.342	3.918	4.587
Average clustering	0.120	0.156	0.155

We analyzed three outages that occurred January 21, January 23, and January 26–27, 2019. We refer to “disappeared” edges as those that existed in the period before each event (January 14–20, January 22, and January 24–25) and did not exist during the outage. Then, we refer to “added” edges as those that existed during the event but not immediately before. These networks are visualized in Fig. 2 and 3 and the measurements of these pairs of networks are given in Table 3.

By comparing the “added connections” and “disappeared connections” networks, we see that each time an outage occurred, novel connections formed during the event had higher clustering and shorter paths between users. This shows that the social structure during outage events had closer connections among the users, especially in network subcommunities, potentially pointing to unifying ties that can be induced by external events. However, over time and with each additional outage event, each “added” graph had longer mean shortest path lengths than the one before it, as well as more connected components and a larger number of separate communities. The unifying effects of the events potentially weakened over time during repeated events or the more severe nature of the last outage (a full internet outage) caused a disruption of the closer ties among users. We can also see that conversations inside and outside of Venezuela became more segregated as we see a separation in the networks between blue and red nodes, indicating that the location barrier became more difficult to cross as time went on.

### Measuring information recurrence during internet outages

We analyzed the recurrence patterns of information across language and location communities, focusing on named entities and hashtags as trackable units of information.

### How do prominent named entities and hashtags recur over time in different communities during repeated outage events?

To gather the set of named entities to analyze, we leveraged the state-of-the-art AllenNLP named entity recognition (NER) tool^42^ to identify people, organizations, and locations mentioned in English and Spanish tweets. For Spanish tweets, we retrained the AllenNLP NER model on the WikiNER dataset^43^. We also extracted the hashtags from both English and Spanish tweets. After extracting these units of information, we translated the most common from Spanish to English and vice versa and performed co-reference resolution in order to consolidate equivalent information units that refer to the same entity. For example, “the United States,” “Estados Unidos,” “EEUU,” and “USA” were all merged into a single unit of information to track discussion of the entity across multiple languages. Some of the most common units of information were mentions of “censura” (128k tweets), “internet” (112k tweets), “nicolas maduro” (74k tweets), “medios” (57k tweets), and “juan guiado” (47k tweets); while the least common were “plaza de chacao” (1.4k tweets), “constitucion venezolana” (1.4k tweets), “@monterogabriela” (1.4k tweets), “don ramon” (1.4k tweets), and “2-Feb” (1.0k tweets). A total of 78 different units of information were used for the analysis.

**Figure 4:**
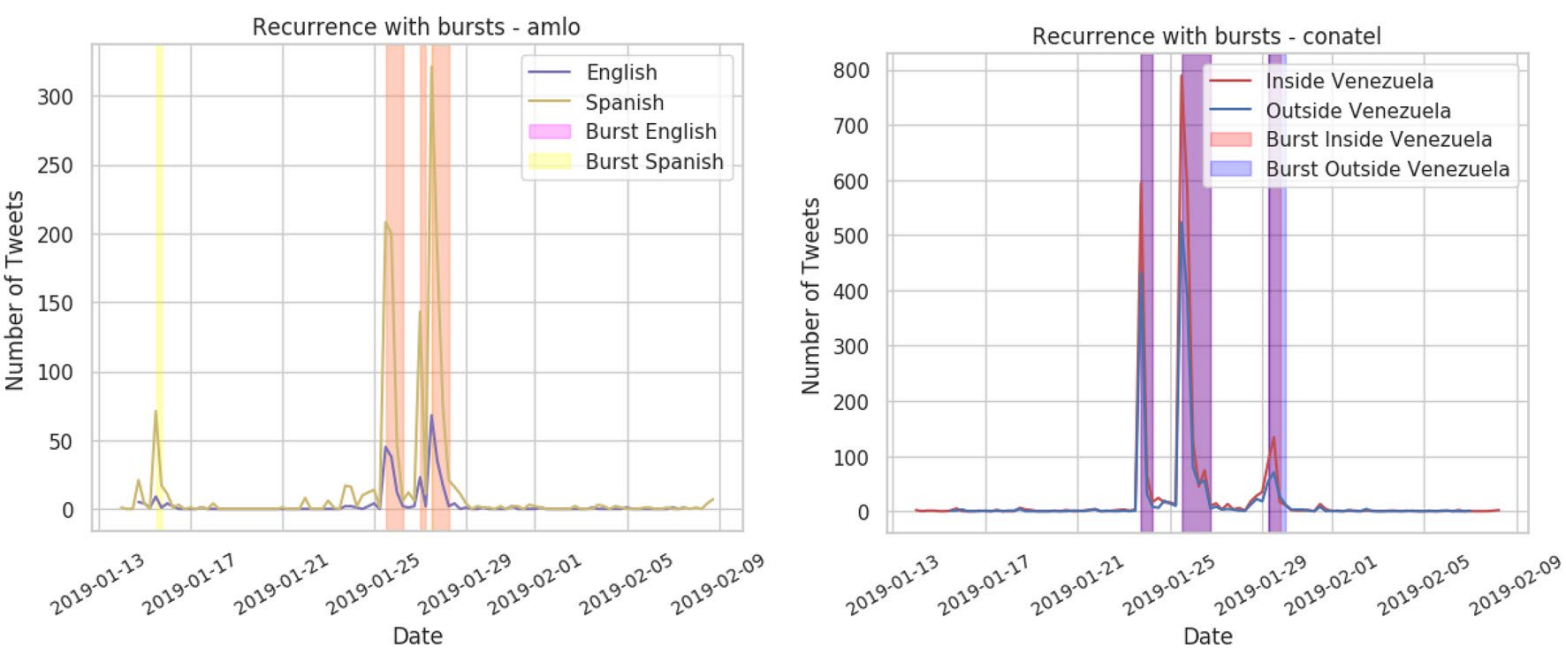
Time series of tweet counts related to specific example units of information with detected burst periods highlighted. The time series are broken down by language (left) and user location (right). For language, we show how “AMLO” (the nickname for the President of Mexico, Andrés Manuel López Obrador) bursts across Spanish Twitter (yellow) and English Twitter (purple), with orange bars indicating simultaneous bursts in both communities. For location, we show how “Conatel” (the National Commission of Telecommunications in Venezuela) bursts inside (red) and outside (blue) of Venezuela, with purple bars indicating simultaneous bursts in both communities.

**Table 4:** The percentage of bursts across all information units that are isolated to each specific community.

Community	Percent of bursts isolated to that community
Spanish	17.0%
English	12.6%
Venezuela	9.00%
Not Venezuela	26.25%

Overall, the majority of bursts cross community lines. Very few bursts occur only in the within-Venezuela community while more than a quarter occur only outside Venezuela.

After identifying tweets that contained prominent named entities and hashtags, we leveraged the Kleinberg algorithm^44^ to identify “bursts” or periods of elevated tweeting activity related to each unit of information. We first separated tweets by user community (English versus Spanish speaking, inside versus outside Venezuela) and constructed activity time series for each information unit for each community separately. We performed burst detection on these community-level time-series and identified cases where a unit of information was bursting only in one community versus bursting in both simultaneously. The examples of bursting behavior for several named entities is presented in Fig. 4. We found that it was more common for a given unit of information to burst simultaneously across languages and locations, meaning that these divisions did not appear to be strong barriers to information spread. The percentage of bursts isolated to the Spanish-speaking community was 17%, to the English-speaking community was 12.6%, to the inside Venezuela community was 9.00%, and to the outside Venezuela community was 26.25%. While most bursts appeared in both communities, topics bursting outside of Venezuela were less likely to also reach inside Venezuela compared with co-bursting rates between other community pair directions. Conversations that were taking place inside Venezuela were also being discussed outside, but those outside the country were less likely to cross into the country as well, as seen in Table 4. This may be because residents of countries outside of Venezuela are discussing their own reactions to censorship events including local context and named entities into their discussion.

### Identifying central users and gatekeepers across communities during outage events

To understand why information sometimes spreads to multiple communities and sometimes only within a single community in the presence of external shock, we analyzed the behaviour of bridging users who formed connective ties between communities using several measures of centrality and gatekeeping behavior. Centrality information is given in Table 5.

### Which users serve in central roles connecting different groups and communities during internet outage events?

**Table 5:** The mean and median inter-community centrality of users in different communities.

Community	Average centrality	Median centrality	Number of users
Spanish	2051.0	24.8	27,071
English	19,869.0	583.8	742
Bilingual	11,304.0	1868.2	6955
Venezuela	2878.0	143.6	9966
Not Venezuela	2988.0	172.3	13,694

The centrality of the Spanish-speaking, English-speaking, and bilingual users is measured across the Spanish- and English-speaking communities, while the centrality of the inside-Venezuela and outside-Venezuela users is measured between the inside and outside Venezuela communities.

**Figure 5:**
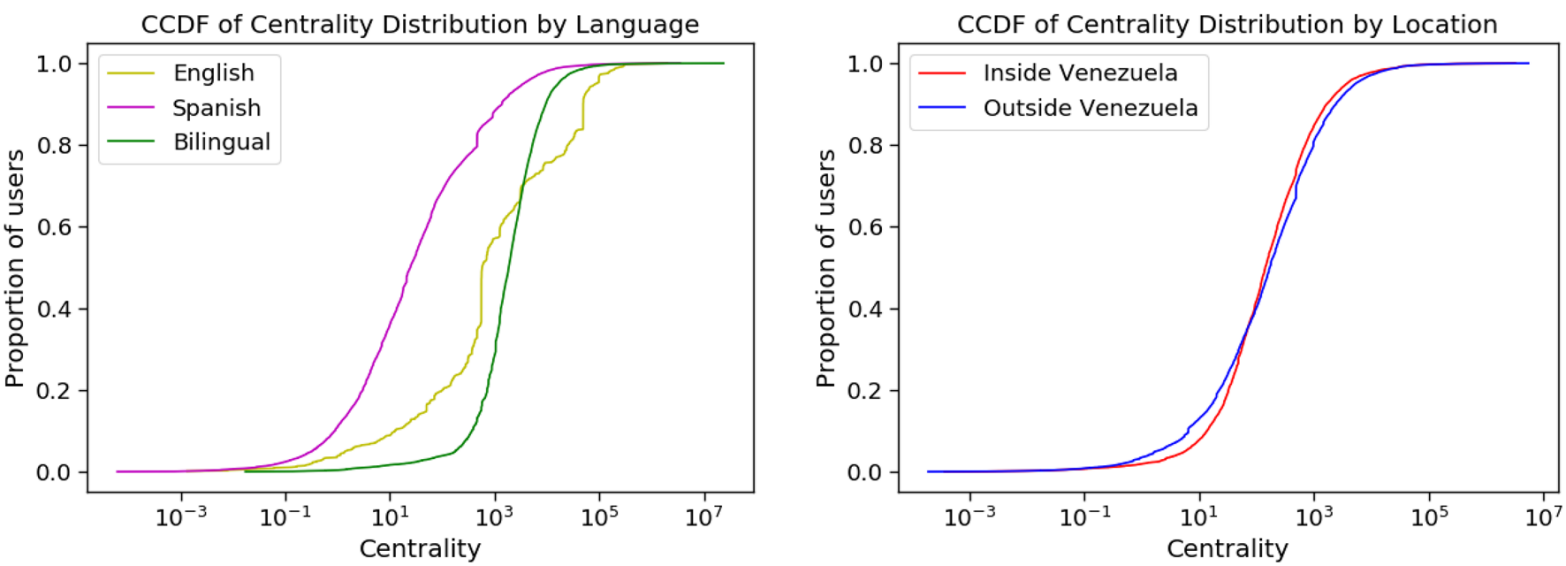
CCDF plots of the inter-community centrality of users from different language (left) and location (right) communities. We observe that language plays a much bigger role in determining user centrality than location does.

To identify these connective users, we rely on betweenness centrality, a measure which identifies users that are commonly found on the shortest path between other users in the network. Rather than measuring the global centrality of the users, we measure it with respect to the shortest paths between users across a pair of communities. Users with a high value of this measure likely have strong connections to both communities and form a social tie between the two communities. We present CCDFs of user centrality between both the English- and Spanish-speaking communities and the inside and outside Venezuela communities in Fig. 5.

We observe that bilingual and English-speaking users are more central across communities than Spanish-speaking users, with these differences being significant with $p < 0.001$ according to a Mann-Whitney U test. Recall that bilingual users are those who have tweeted at least once in English and in Spanish. Bilingual users, who have the highest median centrality, function as bridges across Spanish and English communities. Meanwhile, English speakers have higher average centrality. In this particular dataset, it appears that the impact of speaking a language that is a significant minority in the population leads to high measures of centrality, when centrality is measured as the connection between two communities. This might be because the events are taking place in a Spanish-speaking area and interested English speakers are more likely to interact with the Spanish speakers than vice versa. The Spanish speakers might be more committed to talking with each other as they are experiencing the event and might not look to form connections outside their community. We found that users with the highest centrality between the Spanish- and English-speaking communities included U.S. politician Marco Rubio, Venezuelan journalists Ibéyise Pacheco, American political activist Ajamu Baraka, and NetBlocks, a non-governmental organization that monitors cybersecurity and the governance of the internet.

When we analyze centrality of users located inside Venezuela and those outside Venezuela, we find that some of the same users with high centrality between languages also have high centrality between locations. These users include Marco Rubio, Ibéyise Pacheco, and NetBlocks. Other high centrality users include Venezuelan television commenter Nelson Bocaranda and Venezuelan journalist Milagros Socorro. We find that individuals from both Venezuela and outside Venezuela appear to have similar distribution of centrality measures. This indicates that users both inside and outside the country are providing key links between the two location-based communities rather than a more restrictive bottleneck existing through specific users in one of the communities. Additionally, if we compare the distribution of centrality values between the language and location communities, we see that the centrality of the English-speaking and bilingual users typically exceeds the centrality of the users in the location-based communities. This provides additional evidence that the flow of information between the language-based communities is more restricted to a smaller set of users than for the location-based communities.

### Which users function as gatekeepers across communities and determine if information will spread in a specific community?

**Figure 6:**
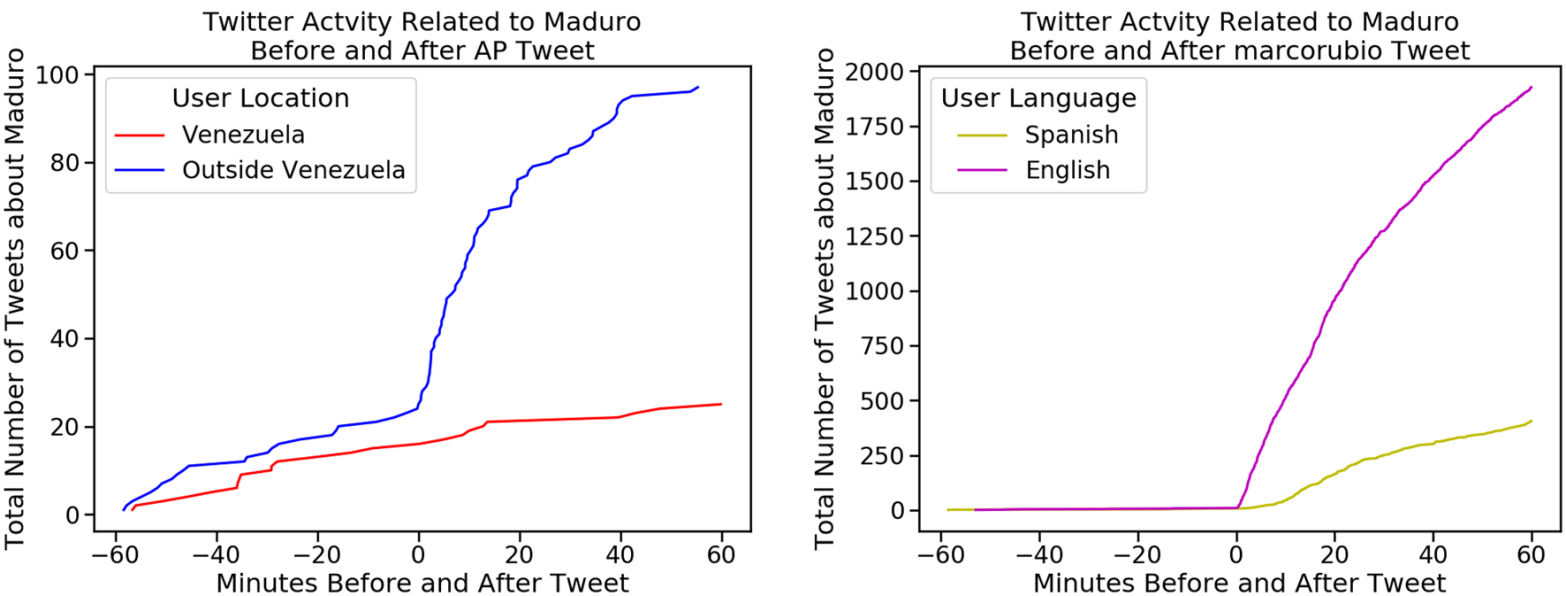
We show examples of Twitter activity within different communities related to Nicolás Maduro following a mention of him by a specific user. As measured from the distance to the line of best fit as shown in Fig. 7, the Associated Press (left) is in the 99.64% percentile for stronger effect outside Venezuela, and Marco Rubio (right) is in the 99.99% percentile for stronger effect in English. We see that after each of their tweets, there is increased spread in each of the communities they influence, which suggests gatekeeping properties in each group.

Rather than identifying global gatekeepers, as has been done in previous work, we attempt to discover which individuals have the largest effect on information being spread to a specific community. To do this, we measure the rate of change of information spread in various communities after a user tweets. We assume that if information spreads more quickly in one community than the contrasting community after a user tweets, then that user may be a gatekeeper to that community (see the example spread in Fig. 6). We then seek to validate this measure by observing its relationship with the bursting patterns of information within individual communities.

**Figure 7:**
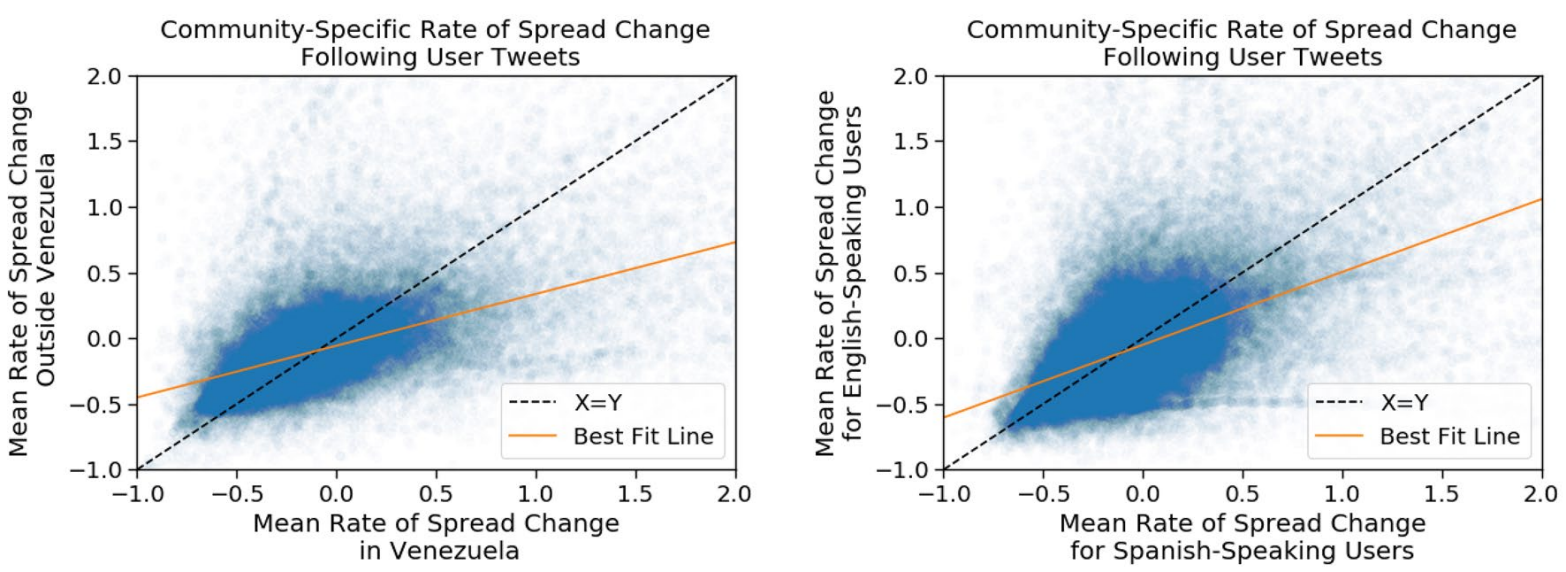
Relationship between the change in rate of spread within each pair of communities. (Left) Comparison of the change in spread rate induced by each user in the outside- versus inside-Venezuela community. (Right) Comparison of the change in spread rate induced by each user in the English- versus Spanish-speaking community. The best fit lines shown are determined using Huber regression and the fits are significant with $p < 0.001$.

When a user *u* tweets about a unit of information *i*, we can measure how quickly *i* spreads throughout each community *c* before and after the tweet. For each *c*, we compute the rate of spread before, $r_b$, and the rate of spread after, $r_a$. We measure the change in rate of spread for the community $\Delta r$ as $\frac{r_a - r_b}{r_b}$. To account for multiple tweets from each user and to increase the robustness of the measure, we average the observed change in rate $\overline{\Delta r}$ across all tweets from the user. To reduce the impact of noise on this analysis, we do not consider the first or last 5% of tweets in the data for a unit of information, ensuring that our statistics have enough data points on either side of the event. To compare the change in rate for each given user between two contrasting communities, we plot $\overline{\Delta r}_\text {comm1}$ on the *x* axis and $\overline{\Delta r}_\text {comm2}$ on the *y* axis. Using this scatter plot, we find the line of best fit $\ell$ to measure the typical relationship between the change in rate in the two communities as shown in Fig. 7. In both cases, these fit lines are significant with $p < 0.001$. To find potential gatekeepers, we find the users that lie the furthest from $\ell$, who appear to have an outsized influence on one community over another.

**Figure 8:**
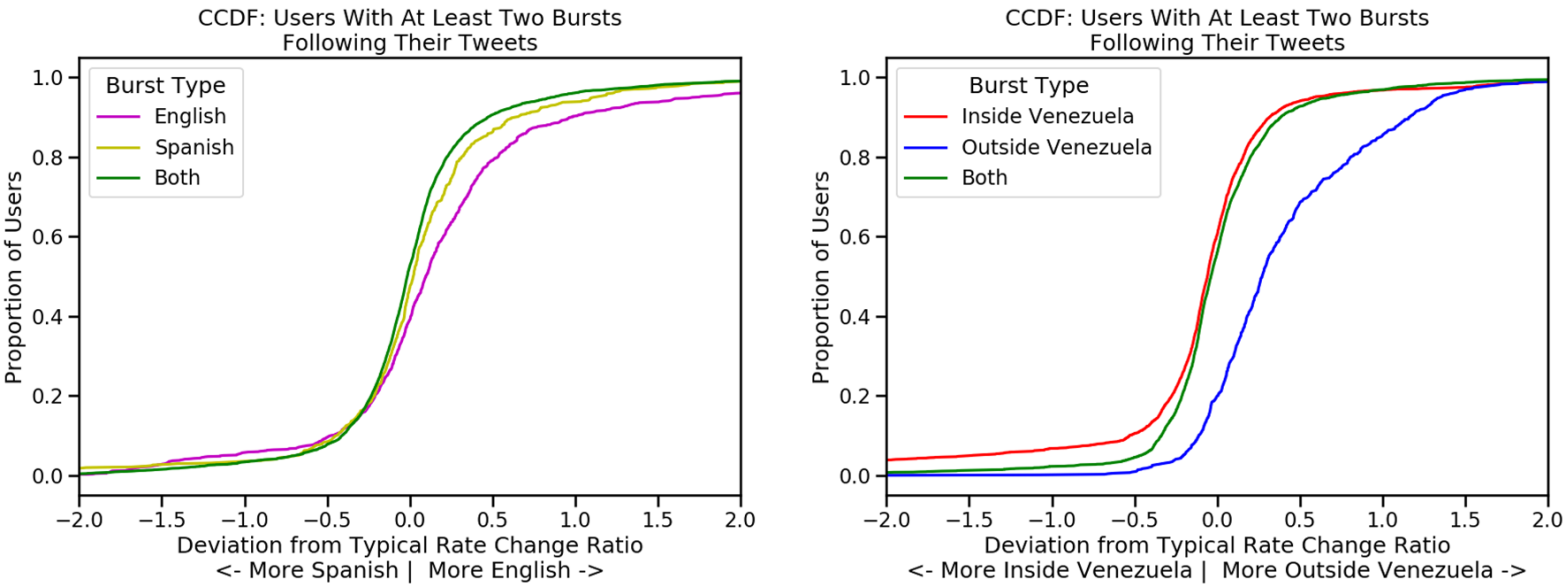
CCDF of the user deviations from the typical ratio of rate changes between communities (distance from the best fit line in Fig. 7) for users with at least two burst-preceding tweets for bursts occurring (left) only in the Spanish-speaking community (yellow), only in the English-speaking community (purple), or across both communities (green); and (right) only in the inside-Venezuela community (red), only in the outside-Venezuela community (blue), or across both communities (green).

**Figure 9:**
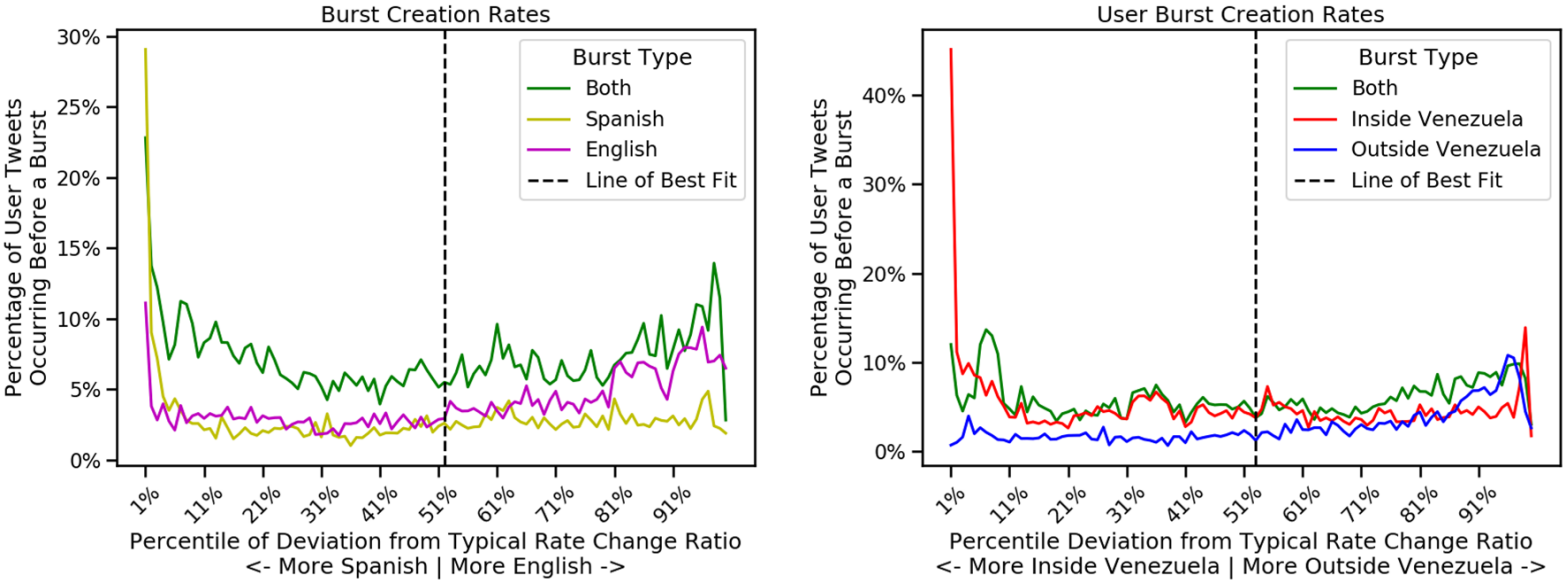
We put our users into percentile bins based on their distance from the typical ratio of rate change within each pair of communities (their distance the line of best fit in Fig. 7). For each group of users by percentile bin, we show the mean percent of these users’ tweets that precede bursts of each type (single community or across communities).

To find additional evidence of gatekeeping behavior, we look for correlations with bursts occurring in a particular community after these users tweet. For each burst regarding a specific unit of information, we identify all tweets related to the same unit of information that occurred in the hour before the burst started or the first hour of the burst and count the number of such tweets for each user. We find that users who have multiple such burst-preceding tweets are also further from $\ell$. If the user tweets before multiple bursts appearing only inside Venezuela, that user also tends to deviate from $\ell$ on the Venezuela side of the line. This relationship is shown in Figs. 8 and 9 providing an indication that the topics these users choose to tweet influence which information spreads within the specific community. As we see in Fig. 8, potential gatekeepers, identified as users with multiple burst-preceding tweets, have a greater distance from the line of best fit in the direction corresponding to the specific location or language community. In Fig. 9, we see that as a user’s tweets venture further from the line of best fit, it is more likely that a community-specific burst will happen after that user tweets about a topic, especially at the far extremes of distance from the typical rate change ratios.

## Discussion

This work presents novel findings on inside and cross-community communication patterns during external shock events by analyzing changes in the network structure, information recurrence, and identifying users who had central or gatekeeping roles across language-specific and location-specific communities during multiple internet outage events in Venezuela in January 2019. We describe specific findings related to each research question below.

### How do external outage events affect social network structure?

We observed that during outage events interaction with new connections formed in networks have shorter mean paths between users and a more clustered structure compared with the connections that are no longer observed. The clustering coefficient, density, and number of communities and connected components of the newly formed connections tend to increase as more outage events occur subsequently. We hypothesize that discussions about the ongoing censorship events generate closer bonds among users as they reach out to spread information about the event. The increase in the mean shortest path length with additional outage events may be related to the increasingly complete nature of the outages. Note, more research is needed focusing on interventions and natural experiments combined with the analysis of a larger quantity of outage events to understand how the specific details of the outage affect the resulting social network structure.

### How do discussions of prominent named entities and hashtags spread over time across communities during repeated outage events?

We found that information bursts tend to cross both language and location community boundaries rather than being limited to a single community. In this sense, social interactions appear to be making users more connected, with people in different communities talking about similar topics. If we compare the co-bursting rates across different communities, we see that information discussed outside the censored country is less likely to also spread inside compared with vice versa. This could be related to the censorship event effectively limiting access to certain information, but it may also be related to individuals tweeting outside the country having different priorities than those inside the country, perhaps talking about censorship as it specifically relates to their country. In contrast, the ongoing censorship within Venezuela became a topic of international interest, causing topics from inside the country to successfully jump to an international audience.

### Which users serve in central roles connecting different groups and communities during outage events?

We found that bilingual users and English speakers had higher centrality than Spanish speakers. This confirms expectations that bilingual users serve as bridges in spreading information across language boundaries. The higher centrality of English-speaking users may be because they are a significant minority in this dataset, with 17.8% of users having English as their primary language. Social connections to the English-speaking portion of the discussion network may be passing through a more limited number of users who are tuned in to the ongoing events. When we analyzed which users performed a central role in connecting the users inside Venezuela with the users outside Venezuela, we found users inside and outside the country had a similar distribution of centrality and lower overall centrality than the high centrality users in the Spanish versus English network. This indicates there is greater overall connectivity between the location-based communities than the language-based communities.

### Which users function as gatekeepers for specific communities, determining whether specific information will spread to that community?

We developed a novel method for identifying gatekeepers that goes beyond the detection of global gatekeepers by comparing the change in rate of information spread across multiple communities. We found that the users who deviate the most from typical levels of rate change also tend to have tweets that precede information subsequently bursting through the community. This shows that not only do these users tend to have an amplifying effect on discussion of specific topics in the short term, but they can also prompt information to burst through the community’s social network. By inference, this means that by choosing whether to tweet about a given topic, they may determine its likelihood of reaching specific audiences. Therefore, these users may function as conduits that allow or prevent information to cross community boundaries during outage events designed to stifle the spread of information.

### Impacts of our findings

Understanding information spread phenomena (e.g., gatekeeping and recurrence) during censorship events and societal instabilities is extremely important. This work provides a novel in-depth analysis of how information diffuses during internet outage events in Venezuela. The same methods can be applied to future internet restrictions in other countries to see if similar conclusions can be reached e.g., Belarus in 2020^45^.

We find that users become more connected during censorship events, with a social network that has higher clustering coefficient, density, and number of connected components and communities. We empirically show that prominent named entities and hashtags tend to burst simultaneously across community boundaries, indicating that censorship events to do not completely inhibit the spread of such information. However, these units of information do co-occur at different rates within various language and location communities, showing there are some limits to the cross-community information flow. We further extend earlier findings about bilingual users serving as bridges across multilingual communities and show the impact of being bilingual on user centrality, a measure of influence levels on how information spreads across communities. We see that language appears to cause greater separation between users than location. While individuals in different locations seem to discuss separate topics, users across multiple locations can serve central community-bridging roles in the social network. Finally, we presented a novel method to identify gatekeepers who spread information across communication barriers such as language and location.

### Future work

This work has wide applicability in understanding the role of cross-community interactions during external events such as censorship and the findings presented here would be strengthened by further analysis of such related occurrences. In the current analysis, we focused only on the spread of text-based pieces of information. However, this analysis can be easily applied to other modalities such as images and videos. For instance, we can analyze how memes spread across languages and locations and observe how different cultures use the same images to communicate different ideas. Additionally, we have presented a method that can identify which users are most likely to spread information across a language or location barrier, but further work is needed to understand the characteristics and behaviors of such users.

## References

[1] Collins L (2017). Gatekeepers of our lives [internet censorship]. Eng. Technol..

[2] Clark, J. D. *et al.* The shifting landscape of global internet censorship (2017).

[3] Wright, J., Darer, A. & Farnan, O. *Automated discovery of internet censorship by web crawling* (Association for Computing Machinery, 2018).

[4] King G, Pan J, Roberts ME (2014). Reverse-engineering censorship in china: Randomized experimentation and participant observation. Science.

[5] Bamman, D., O’Connor, B. & Smith, N. Censorship and deletion practices in Chinese social media. *First Monday* **17** (2012).

[6] Netblocks. Major internet disruptions in venezuela amid protests. https://netblocks.org/reports/major-internet-disruptions-in-venezuela-amid-protests-4JBQ2kyo.

[7] Netblocks. Social media outage and disruptions in venezuela amid incident in caracas. https://netblocks.org/reports/venezuela-social-media-restricted-amid-caracas-incident-zgBLoXA4.

[8] Netblocks. Evidence of regional internet blackouts across venezuela. https://netblocks.org/reports/venezuela-total-internet-blackouts-qr8VeYy5.

[9] Press, T. A. Venezuelan opposition targeted by internet censors (2019). https://www.nbcnews.com/news/latino/venezuelan-opposition-targeted-internet-censors-n966356.

[10] Bolgov, R., Filatova, O. & Semenova, E. Social media in mexico, argentina and venezuela: legal and political framework. In *2017 Conference for E-Democracy and Open Government (CeDEM)* 253–259 (IEEE, 2017).

[11] House, F. Venezuela—freedom house. https://freedomhouse.org/country/venezuela/freedom-net/2019.

[12] Goel, S., Watts, D. J. & Goldstein, D. G. The structure of online diffusion networks. In *Proceedings of the 13th ACM conference on electronic commerce* 623–638 (2012).

[13] Valente TW (2012). Network interventions. Science.

[14] Vespignani A (2009). Predicting the behavior of techno-social systems. Science.

[15] Girvan M, Newman ME (2002). Community structure in social and biological networks. Proc. Natl. Acad. Sci. USA.

[16] Weng L, Menczer F, Ahn Y-Y (2013). Virality prediction and community structure in social networks. Scientific reports.

[17] Bond RM (2012). A 61-million-person experiment in social influence and political mobilization. Nature.

[18] Centola D (2010). The spread of behavior in an online social network experiment. Science.

[19] Borge-Holthoefer J, Baños RA, González-Bailón S, Moreno Y (2013). Cascading behaviour in complex socio-technical networks. J. Complex Netw..

[20] Luarn P, Chiu Y-P (2016). Influence of network density on information diffusion on social network sites: The mediating effects of transmitter activity. Inf. Dev..

[21] Henry D, Stattner E, Collard M (2017). Social media, diffusion under influence of parameters: Survey and perspectives. Procedia Comput. Sci..

[22] Cheng, J., Adamic, L. A., Kleinberg, J. M. & Leskovec, J. Do cascades recur? In *Proceedings of the 25th International Conference on World Wide Web* 671–681 (International World Wide Web Conferences Steering Committee, 2016).

[23] Shin J, Jian L, Driscoll K, Bar F (2018). The diffusion of misinformation on social media: Temporal pattern, message, and source. Comput. Hum. Behav..

[24] Barzilai-nahon, K. Toward a theory of network gatekeeping: A framework for exploring information control. *JASIST* 1493–1512.

[25] Garimella, K., De Francisci Morales, G., Gionis, A. & Mathioudakis, M. Political discourse on social media: Echo chambers, gatekeepers, and the price of bipartisanship. In *Proceedings of the 2018 World Wide Web Conference* 913–922 (International World Wide Web Conferences Steering Committee, 2018).

[26] Welbers K, Opgenhaffen M (2018). Social media gatekeeping: An analysis of the gatekeeping influence of newspapers public facebook pages. New Media Soc..

[27] Kitsak M (2010). Identification of influential spreaders in complex networks. Nat. Phys..

[28] Bakshy, E., Karrer, B. & Adamic, L. A. Social influence and the diffusion of user-created content. In *Proceedings of the 10th ACM conference on Electronic commerce* 325–334 (2009).

[29] Bakshy, E., Hofman, J. M., Mason, W. A. & Watts, D. J. Everyone’s an influencer: quantifying influence on twitter. *Proceedings of the fourth ACM international conference on Web search and data mining* **65–74** (2011).

[30] Jin, H. Detection and characterization of influential cross-lingual information diffusion on social networks. In *Proceedings of the 26th International Conference on World Wide Web Companion* 741–745 (2017).

[31] Cha, M., Haddadi, H., Benevenuto, F. & Gummadi, K. P. Measuring user influence in twitter: The million follower fallacy. In *Fourth international AAAI conference on weblogs and social media* (2010).

[32] Kwak, H., Lee, C., Park, H. & Moon, S. What is twitter, a social network or a news media? In *Proceedings of the 19th international conference on World wide web* 591–600 (AcM, 2010).

[33] Hui C, Goldberg M, Magdon-Ismail M, Wallace WA (2010). Simulating the diffusion of information: An agent-based modeling approach. Int. J. Agent Technol. Syst..

[34] Ali SR, Fahmy S (2013). Gatekeeping and citizen journalism: The use of social media during the recent uprisings in Iran, Egypt, and Libya. Media War Conflict.

[35] West SM (2017). Raging against the machine: Network gatekeeping and collective action on social media platforms. Media Commun..

[36] Bastos MT, Raimundo RLG, Travitzki R (2013). Gatekeeping twitter: Message diffusion in political hashtags. Media Cult. Soc..

[37] Eleta I, Golbeck J (2012). Bridging languages in social networks: How multilingual users of twitter connect language communities?. Proc. Am. Soc. Inf. Sci. Technol..

[38] Eleta, I. Multilingual use of twitter: Social networks and language choice. In *Proceedings of the ACM 2012 conference on Computer Supported Cooperative Work Companion* 363–366 (2012).

[39] Goel, R. *et al.* The social dynamics of language change in online networks. In *International Conference on Social Informatics* 41–57 (Springer, 2016).

[40] Kim, S., Weber, I., Wei, L. & Oh, A. Sociolinguistic analysis of twitter in multilingual societies. In *Proceedings of the 25th ACM conference on Hypertext and social media* 243–248 (2014).

[41] Hale, S. A. Global connectivity and multilinguals in the twitter network. *Proceedings of the SIGCHI Conference on Human Factors in Computing Systems* **833–842** (2014).

[42] Gardner, M. *et al.* Allennlp: A deep semantic natural language processing platform. *arXiv preprint *arXiv:1803.07640 (2018).

[43] Nothman J, Ringland N, Radford W, Murphy T, Curran JR (2013). Learning multilingual named entity recognition from wikipedia. Artif. Intell..

[44] Kleinberg J (2003). Bursty and hierarchical structure in streams. Data Min. Knowl. Discov..

[45] Netblocks. Internet disruption hits belarus on election day. https://netblocks.org/reports/internet-disruption-hits-belarus-on-election-day-YAE2jKB3.

